# A New Triterpenoid Saponin from *Pulsatilla cernua*

**DOI:** 10.3390/molecules15031891

**Published:** 2010-03-16

**Authors:** Yajuan Xu, Lu Bai, Yonghong Liu, Yue Liu, Tunhai Xu, Shengxu Xie, Yunshan Si, Haiou Zhou, Tonghua Liu, Dongming Xu

**Affiliations:** 1 Academy of Traditional Chinese Medicine of Jilin Province, Changchun 130021, China; E-Mail: xyj6492@sohu.com (Y.X.); 2 Department of Traditional Chinese Medicine Chemistry, School of Traditional Chinese Medicine, Beijing University of Chinese Medicine, Beijing 100102, China; 3 Tianjin University of Traditional Chinese Medicine, Tianjin 300193, China; 4 Key Laboratory of Marine Bio-resources Sustainable Utilization, South China Sea Institute of Oceanology, Chinese Academy of Sciences, No. 164 West Xingang Road, Guangzhou 510301, China; E-Mail: yonghongliu@scsio.ac.cn (Y.L.)

**Keywords:** ranunculaceae, *Pulsatilla cernua*, triterpenoid saponin

## Abstract

A new triterpenoid saponin was isolated from *Pulsatilla cernua*, along with eight known triterpenoids and triterpenoid glycosides. The new compound was identified as 3-*O*-*β*-d-glucopyranosyl-(1→4)-*α*-l-arabinopyranosyl-bayogenin-28-*α*-l-rhamnopyranosyl-(1→4)-*β*-d-glucopyranosyl-(1→6)-*β*-d-glucopyranosyl ester (**1**) on the basis of 1D, 2D-NMR techniques, including COSY, HMBC, and HMQC correlations, MS analysis, as well as chemical methods.

## 1. Introduction

*Pulsatilla cernua* (Thunb.) Bercht. et Opiz is widely distributed in Northeast China. The roots are used as a Traditional Chinese Medicine (TCM) for the treatment of amoebic and bacterial dysentery. Phytochemical studies on this plant were reported previously [1,2,3]. In the search for new and bioactive components from TCM, we investigated the roots of *P. cernua*. In the present paper, we report the isolation and structure elucidation of a new triterpenoid saponin from this source.

Our investigation on the constituents in the ethanolic extract of the plant led to the isolation of a new triterpenoid saponin **1** along with eight known constituents: 3-oxo-hederagenin (**2**) [4], hederagenin (**3**) [5], 3-*O*-α-l-arabinopyranosylhederagenin (**4**) [6], 3-*O*-*β*-d-glucopyranosyl-(1→4)-*α*-l-arabinopyranosylhederagenin (**5**) [7], 3-*O*-*β*-d-glucopyranosyl-(1→2)-*α*-l-arabinopyranoside-hederagenin (**6**) [8], 3-*O*-*α*-l-arabinopyranosyl-28-*O*-*α*-l-rhamnopyranosyl-(1→4)-*β*-d-gluco-pyranosyl-(1→6)-*β*-d-glucopyranosylhederagenin (**7**) [9], 3-*O*-*β*-d-glucopyranosyl-(1→4)-*α*-l-arabino-pyranosyl-28-*O*-*α*-l-rhamnopyranosyl-(1→4)-*β*-d-glucopyranosyl-(1→6)-β-d-glucopyranosyl-hedera-genin (**8**) [10] and 3-*O*-*β*-d-glucopyranosyl(1→2)-*β*-d-glucopyranosylhederagenin-28-*O*-*α*-l-rhamno-pyranosyl-(1→4)-*β*-d-glucopyranosyl-(1→6)-*β*-d-glucopyranosyl (**9**) [11], The compounds **2**, **4**, **5**, **7**-**9** were isolated for the first time from *P. cernua*. Herein, we describe the isolation and structure elucidation of the new compound **1**. 

## 2. Results and Discussion

Compound **1**, obtained as a white amorphous powder, turned red upon coloration with Liebermann-Burchard reagent. The molecular formula C_59_H_96_O_28_ was determined by HRESIMS which exhibited a quasi-molecular ion peak at *m/z* 1251.6032 [M-H]^­^ (calcd. for [C_59_H_96_O_28_-H]^­^ 1251.6010). On acidic hydrolysis, **1** afforded sugar moieties that were identical to authentic samples of arabinose, rhamnose, and glucose. The six tertiary methyl groups [δ 0.85, 0.86, 1.00,1.08, 1.14, and 1.18 (each 3H, s)] , one trisubstituted olefinic proton δ 5.41 (1H, t-like, H-12), and a signal at δ 3.15 (1H, dd, *J* = 3.0, 12.5 Hz, H-18) observed in the ^1^H-NMR spectrum coupled with the information from the ^13^C-NMR spectrum (six methyl group carbons at δ 15.1, 17.5, 17.8, 24.1, 26.7, and 33.2, and two olefinic carbons at δ 123.4 and 144.2) indicated that the aglycone possessed an olean-12-ene skeleton. Comparison of the ^13^C-NMR data of this aglycone (Table 1) with those of bayogenin (2*α*,3*β*,23-trihydroxyolean-12-en-28-oic acid) [5], showed that the signal for C-3 of **1** was shifted significantly downfield by +4.4 ppm to 83.0, and the C-28 signal was shifted upfield by –2.0 ppm to 176.6, while the other signals were almost identical, indicating that the aglycon of **1** was indeed bayogenin. Its ESI-MS spectrum showed a quasi-molecular ion at *m/z* 1251 [M-H] ^­^, confirming a molecular weight of 1252. An ion at *m/z* 781 [M-H-470 (162+162+146)]^­^, taken as evidence for the direct elimination of two hexoses and one deoxyhexose, indicated the presence of a trisaccharide unit at C-28 of the aglycone, because the ester glycosidic linkage was more easily broken than the *O*-glycosidic linkage. In addition, the intense ion peaks at *m/z* 619 [M-H-470-162]^­^, 487 [M-H-470-162-132]^­^, suggested the presence of disaccharide unit including arabinose and glucose at C-3 of the aglycone. The ^13^C-NMR signals due to sugar moieties were almost superimposable on those of 3-*O*-*β*-d-glucopyranosyl-(1→4)-*α*-L–arabino-pyranosylhederagenin-28-*α*-l-rhamnopyranosyl-(1→4)-*β*-d-glucopyranosyl-(1→6)-*β*-d-glucopyrano-syl ester [10]. In a comparison of the ^13^C-NMR signals for sugar moieties of **1** with those of the known saponin of leontoside (3-*O*-*β*-d-glucopyranosyl-(1→4)-*α*-l-arabinopyranosylhederagenin-28-*α*-l-rhamnopyranosyl-(1→4)-*β*-d-glucopyranosyl-(1→6)-*β*-d-glucopyranosyl ester) [10], all signals due to the sugar moieties of **1** were almost superimposable with those of leontoside, indicating the sugar moieties of **1** was same as those of the latter, so the 3-hydroxy and 28-carbonyl groups carried the same disaccharide chain and trisaccharide chain, respectively. Consequently, compound **1** should be a bisdesmosidic saponin in which the disaccharide chain of arabinose and glucose was bound to the aglycone by a glycosidic linkage at C-3, while a trisaccharide chain of glucose, glucose, and rhamnose was bound by a glycosidic ester linkage at C-28. The ^1^H- and ^13^C-NMR spectrum of **1** exhibited five sugar anomeric protons at δ 4.91 (1H, d, *J* = 7.0 Hz, ara H-1), 5.22 (1H, d, *J* = 8.0 Hz, glc H-1), 6.21(1H, d, *J* = 8.0 Hz, glc′ H-1′), 4.91 (1H, d, *J* = 7.9 Hz, glc′′ H-1′′), 5.82 (1H, br s, rha H-1) and carbons at δ 95.8, 102.9, 105.0, 106.8, and 107.3 (Table 1). The methyl carbon signal at δ 18.7 and the doublet methyl proton signal at δ1.71 (3H, d, *J* = 6.0 Hz, rha H-6) indicating the presence of one 6-deoxysugar. These coupling constants indicated that the glycosidic linkage of arabinose, rhamnose were *α* configuration, and those of glucose were *β* configuration [12,13]. The ^1^H- and ^13^C-NMR signals for the aglycone and sugar moieties of **1** was assigned based on the 1D and 2D-NMR spectra (COSY, DEPT, HMQC, and HMBC). The sugar arrangements were determined to be 3-*O*-*β*-d-glucosyl-(1→4)-*α*-l-arabinose by the HMBC which showed the correlations between H-1 of glc at δ 5.22 and C-4 of ara at δ 80.4, between H-1 of ara at δ 4.91 and C-3 of aglycone at δ 83.0, and 28-*α*-l-rhamnosyl (1→4) -*β*-d-glucosyl (1→6)-*β*-d-glucose by the HMBC which showed the correlations between H-1 of rha at δ 5.82 and C-4 of glc′′ at δ 78.9, between H-1 of glc′′ at δ 4.91 and C-6 of glc′ at δ 69.4, between H-1 of glc′ at δ 6.21 and C-28 of the aglycone at δ 176.6. On the basis of these evidences, **1** was identified as 3-*O*-*β*-d-glucopyranosyl-(1→4)-*α*-l-arabinopyranosylbayogenin-28-*α*-l-rhamnopyranosyl-(1→4)-*β*-d-glucopyranosyl-(1→6)-*β*-d-glucopyranosyl ester.

**Figure 1:**
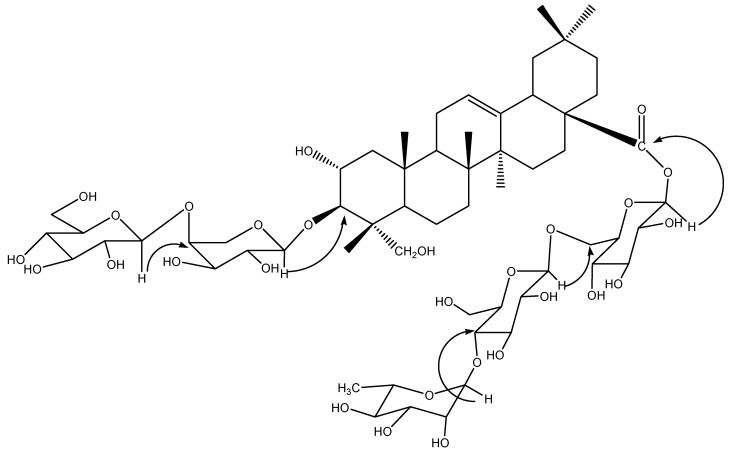
Structure and key HMBC correlations (H → C) of compound **1**.

## 3. Experimental 

### 3.1. General

The melting point was determined on a Kofler-microscope apparatus and is uncorrected. The IR spectra were measured on a Y-Zoom scroll Fourier Transform infrared (FTIR) spectrometer using KBr discs. The ESI-MS was recorded on LCQ-1700 ESI-MS instrument made by the Finnigan (USA). The NMR spectra were obtained on Bruker AM-500 instrument, using TMS as internal standard. HPLC (600E HPLC, Waters, USA) was performed using a ODS column (Shim-park PREF-ODS, 250 × 4.6 mm). Column chromatography was performed on silica gel (200-300 mesh, Qingdao Oceanic Chemical Industry China) and ODS reversed silica gel (250 × 25 mm, Nacalai Tesque, Kyoto, Japan). Macroporous resin D_101_ made in Nankai University, Tianjin. TLC was conducted on silica gel 60 F_254_ (Merck). Spots were detected after spraying with 10% H_2_SO_4_.

### 3.2. Plant Material

The roots of *P. cernua* were collected in August 2007, near Yanji city, Jilin Province, China and identified by Professor Minglu Deng, Changchun College of Traditional Chinese Medicine. A voucher specimen (20070827) was deposited in Academy of Traditional Chinese Medicine and Material Medica of Jilin Province, Changchun, China.

### 3.3. Extraction and Isolation 

The dry roots of *P. cernua* (5 kg) were extracted with 80% EtOH (70–80 °C, 3 × 15 L, 4 h, each). The 80% EtOH solution were heated on steam bath to remove EtOH. The water solution was chromatographed over a D_101_ macroporous resin column (10 × 80 cm), eluted successively with 10% EtOH, 40% EtOH, 80% EtOH. The 40% EtOH eluate and 80% EtOH eluate were evaporated to dryness to give 40% EtOH extract (120 g), and 80% EtOH extract (26 g), respectively. The 40% EtOH crude extract (60 g) was chromatographed on silica gel (1.2 kg, 200 mesh) with CHCl_3_-MeOH-H_2_O [(50:5:1, lower layer → 10:5:1, lower layer), to afford five fractions (fr.1 (1.2 g), fi.2 (0.35 g), fr.3 (0.8 g), fr.4 (0.6 g), fr.5 (0.65 g). Fr.1, fi.2 and fr.4 were dissolved in MeOH to give compounds **2** (1.0 g), **3** (118 mg), and **4** (46 mg), respectively. Fi. 3 was further subjected to silica gel column chromatography [CHCl_3_-MeOH-H_2_O (30:3:1, lower layer → 10:3:1, lower layer→ 6:4:1) → EtOH], to obtain four fractions (fi 4-1-fi 4-5). Fi.4-4 was dissolved in MeOH to give **5** (102 mg). Fi 5 was purified by reversed-phase silica gel column chromatography [MeOH-H_2_O (70:30→90:10) → MeOH] to give **6** (45 mg). The 80% EtOH extract (26 g) was chromatographed on silica gel (0.6 kg, 200 mesh) with CHCl_3_-MeOH-H_2_O [ (50:10:1, lower layer → 10:10:1, lower layer)], to afford four fractions [fr.1 (1.4 g), fi.2 (1.2 g), fr.3 (0.8 g), fr.4 (0.6 g)]. Fi 1 was purified by reversed-phase silica gel column chromatography [MeOH- H_2_O (60:40→90:10) →MeOH] to give two fractions [fr.1 (1.0 g), fi.2 (0.7 g)]. Fi 1 was dissolved in MeOH to give compounds **1** (0.8 g) and **9** (47 mg). Fi.2 was further subjected to silica gel column chromatography [CHCl_3_-MeOH-H_2_O (30:5:1, lower layer →10:5:1, lower layer → 6:4:1) → EtOH], to furnish five fractions (fi 2-1-fi 2-5). Fi.2-2 was dissolved in MeOH to give **7** (65 mg). Fi.2-4 was further subjected to silica gel column chromatography [CHCl_3_-MeOH-H_2_O (30:5:1, lower layer →10:5:1, lower layer→6:4:1) →EtOH] to give **8** (160 mg).

### 3.4. Acid Hydrolysis of **1**

Compound **1** (10 mg) was heated with 2 M HCl-MeOH (10 mL) under reflux for 3h. The reaction mixture was diluted with H_2_O and extracted with CHCl_3_. The water layer was neutralized with Na_2_CO_3_, concentrated, and subjected to TLC analysis with authentic glucose, arabinose, rhamnose, and developed with CH_2_Cl_2_-MeOH-H_2_O (15 ׃6׃1). Detection was carried out with aniline phthalate spray.

### 3.5. Characterization of Compound **1**

White powder, mp 221-223 °C; [α]_D_^20^-37.7° (c 0.30; MeOH); HRESIMS *m/z* found 1251.6032 [M-H] ^­^ (calcd. for [C_59_H_96_O_28_-H]^­^, 1251.6010). ESIMS *m/z* 1251 [M-H]^­^, 781 [M-H-470 (162+162+146)]^­^, 619 [M-H-470-162]^­^, 487[M-H-470-162-132]^­^, ^1^H-NMR (500 MHz, pyridine-d_5_) δ (ppm), *J* (Hz) and ^13^C-NMR (125 MHz, pyridine-d_5_) δ (ppm): see Table 1.

## 4. Conclusions 

A new triterpenoid saponin, 3-*O*-*β*-d-glucopyranosyl-(1→4)-*α*-l-arabinopyranosylbayogenin-28-*α*-l-rhamnopyranosyl-(1→4)-β-d-glucopyranosyl-(1→6)-*β*-d-glucopyranosyl ester (1), was isolated from the roots of from *P. cernua*. Most of the terpenoid saponins are oleane-type (oleanolic acid and hederagenin) from the genus *Pulsatilla*. This is the first time terpenoid saponin with 2*α*-hydroxy in its aglycone (bayogenin) has been isolated from the genus *Pulsatilla*. This finding is meaningful for the chemotoxonomy of this species.

## Figures and Tables

**Table 1:** ^1^H- and ^13^C-NMR spectral data of compound **1** (recorded at 500/125 MHz in pyridine-d_5_; δ in ppm, *J* in Hz).

No.	δc	δ_H_ (*J*, Hz)	No.	δc	δ_H_ (*J*, Hz)
1	44.4		C-3		
2	71.0	4.20	Ara-1	106.8	4.91 (d, 7.0 Hz)
3	83.0	4.24	2	73.7	4.68
4	42.4		3	71.4	4.27
5	47.9		4	80.4	4.80
6	18.1		5	65.4	4.48, 4.39
7	33.0		Glc-1	107.3	5.22 (d, 8.0)
8	40.2		2	76.0	3.92
9	48.7		3	78.9	4.15
10	37.2		4	71.0	4.16
11	23.8		5	78.6	3.89
12	123.4	5.41 (t-like)	6	62.6.	4.29，4.45 (d, 7.6)
13	144.2		C-28		
14	42.9		Glc-1′	95.8	6.21 (d, 8.0)
15	28.4		2′	74.2	4.10
16	23.5		3′	78.2	4.19
17	46.3		4′	70.4	4.25
18	41.8	3.15 (dd, 3.0, 12.5)	5′	78.3	4.08
19	47.2		6′	69.4	4.29，4.62
20	30.9		Glc-1′′	105.0	4.91 (d, 7.9)
21	34.1		2′′	75.5	3.89
22	32.7		3′′	76.7	4.11
23	65.4	3.67 (d, 10.0) 4.54	4′′	78.9	4.34
24	15.1	1.00 (s)	5′′	77.3	3.81
25	17.5	1.08 (s)	6′′	61.4	4.06, 4.21
26	17.8	1.14 (s)	Rha-1	102.9	5.82 (br, s)
27	26.7	1.18 (s)	2	72.7	4.61
28	176.6		3	72.9	4.50
29	33.2	0.85 (s)	4	74.0	4.27
30	24.1	0.86 (s)	5	70.9	4.85
			6	18.7	1.71 (d, 6.0)
